# Exposure to baby-friendly hospital practices and mothers’ achievement of their planned duration of breastfeeding

**DOI:** 10.1186/s12884-020-02904-0

**Published:** 2020-05-01

**Authors:** Kris Y. W. Lok, Charlotte L. Y. Chow, Heidi S. L. Fan, Vincci H. S. Chan, Marie Tarrant

**Affiliations:** 1 School of Nursing, Li Ka Shing Faculty of Medicine, The University of Hong Kong, 4/F, William MW Mong Block, 21 Sassoon Road, Pokfulam, Hong Kong; 2 School of Nursing, University of British Columbia, 1147 Research Road, ART 360B, Kelowna, BC Canada

**Keywords:** Baby-friendly hospital practices, Breastfeeding, Chinese, Intended duration

## Abstract

**Background:**

Both breastfeeding intentions and exposure to baby-friendly hospital practices were found to be associated with a longer duration of breastfeeding. This study aims to examine the effect of exposure to baby-friendly hospital practices on mothers’ achievement of their planned duration of breastfeeding.

**Methods:**

A total of 1011 mother-newborn pairs from the postnatal units of four public hospitals in Hong Kong were recruited. Sociodemographic data and breastfeeding intention data were collected via self-report questionnaires during the postnatal hospitalization and exposure to Baby-Friendly hospital practices were assessed through hospital records and maternal self-report. Breastfeeding status after hospital discharge was assessed through telephone follow-up for up to 12 months postnatal, or until participants were no longer breastfeeding.

**Results:**

Only 55% (*n* = 552) of study participants achieved their intended duration of breastfeeding. Participants with higher socioeconomic status, previous breastfeeding experience, and those who had lived in Hong Kong for less than 5 years, were more likely to achieve their planned duration of breastfeeding. Among baby-friendly hospital practices, feeding only breast milk during the hospitalization and providing information about breastfeeding support on discharge were associated with participants’ achieving their individual breastfeeding intentions. After adjustment, when compared with women who experienced onebaby-friendly practice, participants who experienced six baby-friendly hospital practices were significantly more likely to achieve their planned duration of breastfeeding (adjusted odds ratio = 8.45, 95% confidence interval 3.03–23.6).

**Conclusions:**

Nearly half of participants did not achieve their planned breastfeeding duration. Exposure to baby-friendly hospital practices, especially in-hospital exclusive breastfeeding and providing breastfeeding support information upon hospital discharge may help more mothers to achieve their individual breastfeeding goals.

## Significance statement

*What is already known on this subject?* Both breastfeeding intentions and exposure to baby-friendly hospital practices have been associated with a longer duration of breastfeeding. The effect of baby-friendly practices implemented in Hong Kong on mothers’ ability to meet their individual breastfeeding goals is needed. *What this study adds?* Exposure to baby-friendly hospital practices, especially in-hospital exclusive breastfeeding and providing breastfeeding support information upon hospital discharge may help more mothers to achieve their individual breastfeeding goals.

## Background

Breastfeeding has substantial protective health benefits and reduces the risk of infant morbidity and mortality from a number of infectious diseases [1] and the benefits of breastfeeding, especially exclusive breastfeeding, are well recognized [1, 2]. The World Health Organization and the United Nations Children’s Fund have set a goal to increase breastfeeding rates to 75% in early infancy, to 50% at six months and to 25% at one year of age [3]. To protect, promote, and support breastfeeding, the World Health Organization and United Nations Children’s Fund developed the Baby-Friendly Hospital Initiative (BFHI) as a global program in 1991 [4]. The BFHI is a set of 10 evidence-based practices (10 steps) that hospitals and maternity units should implement to support breastfeeding. Many studies have examined the effect of maternal exposure to “baby-friendly steps” on breastfeeding initiation and duration [5–9]. Thus, a substantive body of evidence shows that implementation of BFHI improves breastfeeding outcomes.

One study examining how non-compliance with each baby-friendly step influenced breastfeeding duration found that exposure to a greater number of baby-friendly steps was associated with longer breastfeeding duration [10]. Other studies have shown that, for new mothers, exposure to more steps is associated with lower risk of cessation and therefore with longer duration of breastfeeding [9]. A recent systematic review found a positive graded association between the number of BFHI steps that women experience in the hospital and earlier breastfeeding initiation, exclusive breastfeeding at hospital discharge and longer breastfeeding duration [5]. Therefore, efforts to increase implementation of the BFHI could be anticipated to result in an increased duration of breastfeeding.

Maternal breastfeeding intentions are also a significant predictor of actual breastfeeding practices [11, 12]. Despite setting breastfeeding goals, many mothers do not achieve their intended duration of breastfeeding. Findings from the Infant Feeding Practices Study II in the United States (US) showed that although 85% of pregnant women intend to exclusively breastfeed, only one-third of mothers (32.4%) achieve their planned duration of exclusive breastfeeding [11]. In Hong Kong, a recent study reported that 78.1% of participants intend to exclusively breastfeed, with a median intended duration of 26 weeks [12]. Population-based data shows that although more than 88% of mothers initiate breastfeeding, one third of infants are still receiving any breast milk at six months of age and only 2% achieve six months of exclusive breastfeeding [13]. Therefore, similar to many developed countries, in Hong Kong breastfeeding duration is short and most new mothers fail to meet their individual breastfeeding goals.

In maternal and child health care settings, a primary goal of practitioners should be to help new mothers achieve their individual breastfeeding goals. In Hong Kong, there has been a recent government directive for the full implementation of the BFHI in all public hospitals over the next five years [14]. Although several studies have examined the effect of baby-friendly practices on mothers’ ability to meet their individual breastfeeding goals [7, 11], the availability of non-US data is limited. Therefore, this study aimed to examine the effect of exposure to baby-friendly hospital practices on mothers’ achievement of their planned duration of breastfeeding.

## Methods

### Study design and participants

The basis for this analysis was a multi-center prospective cohort study that recruited mother-infant pairs (*n* = 1287) from the postnatal obstetric units of four geographically distributed public hospitals in Hong Kong from 2011 to 2012. One thousand and eleven participants reported their intended duration of breastfeeding and were included in the analysis as we used this question to categorize meeting their intended duration of breastfeeding. The remaining 276 participants had missing data for the main variables and were excluded. Participants were all Cantonese-speaking Hong Kong residents who gave birth to a single full-term infant, intended to breastfeed, and had no serious medical or obstetric complications. Participants were excluded if the newborn had a birthweight of < 2500 g, a 5-min Apgar score of < 7, and if they were admitted to the special care nursery for > 48 h after birth or were admitted to the neonatal intensive care unit.

Study participants were recruited by a trained research nurse within the first 24 h after giving birth. In-hospital data collection consisted of a baseline self-administered demographic questionnaire, maternal and newborn data, exposure to six of the ten baby-friendly steps, and in-hospital infant feeding data. We measured the six out of ten baby-friendly steps that directly affect individual mothers including mothers initiating breastfeeding within 1 h of birth (step 4), the hospital giving no food or drink other than breastmilk unless medically indicated (step 6), rooming in (step 7), breastfeeding on demand (step 8), giving no teats (step 9) and providing mothers with information on breastfeeding support (step 10). Maternal and newborn data, exposure to baby-friendly hospital steps, and in-hospital infant feeding data were extracted from the maternal and/or newborn health record by the research nurse and were validated with the participant. Follow-up newborn feeding data were collected over the telephone by a trained study research assistant at 1, 2, 3, 6, and 12 months post-partum or until weaned.

As part of the in-hospital demographic questionnaire, mothers were asked “How many weeks in total do you plan to breastfeed?” At the follow-up telephone interviews, participants were asked “What was the age of your baby (in weeks) when she or he was no longer receiving any breast milk?” We used these two questions to compute a proportion that reflected the achieved intended duration of breastfeeding, with a possible range from 0 to 100%. Participants were then categorized as having achieved their intended breastfeeding duration (yes/no), with those who breastfed for ≥90% of their intended duration categorized as yes and those who breastfed for < 90% as no. Participants still breastfeeding at the end of the 12-month follow-up were also considered to have met their intentions, irrespective of their intended duration of breastfeeding. The main predictor variables were the six baby-friendly hospital steps, and maternal and infant data. Consistent with other studies [8–10], we measured the six out of ten baby-friendly steps that affect individual mothers including: (1) step 4: help mothers to initiate breastfeeding within the first hour after birth; (2) step 6: give newborns no food or drink other than breast milk, unless medically indicated; (3) step 7: practice of rooming-in; (4) step 8: encourage breastfeeding on demand; (5) step 9: give breastfeeding newborns no artificial teats or pacifiers; and (6) step 10: provide mothers with information about breastfeeding support on hospital discharge.

The Institutional Review Board of the University of Hong Kong/Hospital Authority Hong Kong West Cluster and the participating study sites ethics committees approved the study. All participants provided informed written consent.

### Statistical analysis

Descriptive statistics were used to compare the sociodemographic characteristics of participants by achievement of their planned duration of breastfeeding. We used unadjusted and adjusted logistic regression models to examine the effect of baby-friendly hospital steps on participants’ meeting their planned duration of breastfeeding. Model 1 was adjusted for the six BFHI steps only and Model 2 was additionally adjusted for sociodemographic variables. We computed the proportion of participants’ who achieved their planned duration of breastfeeding by how many Baby-friendly hospital practices they experienced, and we used unadjusted and adjusted logistic regression analysis to examine the effect of the number of practices on achieving breastfeeding intentions. In all multiple logistic regression models, the Hosmer–Lemeshow test [15] was used to assess the goodness of fit of the models.

Data were analyzed using Stata version 14.1 statistical software (StataCorp LP, College Station, Texas, USA) [16]. A 5% level of significance was used in all statistical tests.

## Results

A total of 1011 participants were included in this study that reported their intended duration of breastfeeding. The median intended duration of breastfeeding was 26 weeks and only 55% (*n* = 552) of participants achieved their planned duration of breastfeeding. The median difference between intended and actual duration of breastfeeding was 14.5 weeks.

The characteristics of the study participants by achievement of their planned duration of breastfeeding are shown in Table 1. Participants meeting their planned duration of breastfeeding had a higher education level (*p* = .006), lived in Hong Kong for a shorter duration (*p* = .022), were themselves breastfed (*p* < .001), had previous breastfeeding experience (p < .001), were multiparous (p < .001), and had a partner who preferred breastfeeding (p < .001). Exposure to four of the six measured baby friendly hospital practices was significantly associated with achieving breastfeeding intentions (*p* < 0.05).

**Table 1:** Comparison of characteristics by participants’ achievement of their planned duration of breastfeeding (*n* = 1011)

Characteristics	Total N (%) ***N*** = 1011	Did Not Meet Planned Duration N (%) N = 552	Met Planned Duration N (%) ***N*** = 459	***p***-value
Maternal age				.063
18–24 years	63 (6.2)	44 (8.0)	19 (4.1)	
25–29 years	251 (24.8)	139 (25.2)	112 (24.4)	
30–34 years	435 (43.0)	235 (42.6)	200 (43.6)	
≥ 35 years	262 (25.9)	134 (24.3)	128 (27.9)	
Maternal education				.006
Primary	30 (3.0)	18 (3.3)	12 (2.6)	
Secondary	575 (56.9)	337 (61.1)	238 (51.9)	
University degree or above	406 (40.2)	197 (35.7)	209 (45.5)	
Monthly family income (HKD)				.165
< $15,000	175 (17.3)	100 (18.1)	75 (16.3)	
$15,000–$29,999	369 (36.5)	212 (38.4)	157 (34.2)	
≥ $30,000	467 (46.2)	240 (43.5)	227 (49.5)	
Length of Residence in Hong Kong				.022
< 5 years	78 (7.7)	36 (6.5)	42 (9.2)	
5 to ≥15 years	332 (32.8)	167 (30.3)	165 (36.0)	
Since birth	601 (59.5)	349 (63.2)	252 (54.9)	
Returning to work post-partum				.074
No	360 (35.6)	183 (33.2)	177 (38.6)	
Yes	651 (64.4)	369 (66.9)	282 (61.4)	
Planning to exclusively breastfeed				<.001
No	232 (23.0)	150 (27.2)	82 (17.9)	
Yes	779 (77.1)	402 (72.8)	377 (82.1)	
Participant was breastfed				.026
No	591 (58.5)	340 (61.6)	251 (54.7)	
Yes	420 (41.5)	212 (38.4)	208 (45.3)	
Previous breastfeeding experience				<.001
No	621 (61.4)	369 (66.9)	252 (54.9)	
Yes	390 (38.6)	183 (33.2)	207 (45.1)	
Partner’s Infant feeding preference				.038
Breastfeeding	426 (42.1)	217 (39.3)	209 (45.5)	
No preference	509 (50.4)	285 (51.6)	224 (48.8)	
Infant Formula & mixed feeding	76 (7.5)	50 (9.1)	26 (5.7)	
Parity				.008
Primiparous	568 (56.2)	331 (60.0)	237 (51.6)	
Multiparous	443 (43.8)	221 (40.0)	222 (48.4)	
Delivery type				.981
Spontaneous vaginal	799 (79.0)	434 (78.6)	365 (79.5)	
Assisted vaginal	48 (4.8)	26 (4.7)	22 (4.8)	
Planned caesarean	77 (7.6)	43 (7.8)	34 (7.4)	
Emergency caesarean	87 (8.6)	49 (8.9)	38 (8.3)	
Step 4: Breastfeeding initiation within 1 hour				.033
No	548 (54.2)	316 (57.3)	232 (50.5)	
Yes	463 (45.8)	236 (42.8)	227 (49.5)	
Step 6: Only breastmilk given				<.001
No	604 (59.7)	375 (67.9)	229 (49.9)	
Yes	407 (40.3)	177 (32.1)	230 (50.1)	
Step 7: Rooming-in				.893
No	315 (31.2)	171 (31.0)	144 (31.4)	
Yes	696 (68.8)	381 (69.0)	315 (68.6)	
Step 8: Breastfeeding on demand				.773
No	220 (21.8)	122 (22.1)	98 (21.4)	
Yes	791 (78.2)	430 (77.9)	361 (78.7)	
Step 9: No teat given				<.001
No	230 (22.8)	153 (27.7)	77 (16.8)	
Yes	781 (77.3)	399 (72.3)	382 (83.2)	
Step 10: Provide information on breastfeeding support				.007
No	162 (16.0)	104 (18.8)	58 (12.6)	
Yes	849 (84.0)	448 (81.2)	401 (87.4)	

Table 2 presents the associations between the baby-friendly hospital practices and achieving breastfeeding intentions. In the fully adjusted analysis (Model 2), the association between achieving the planned duration of breastfeeding and exposure to baby-friendly hospital practices was statistically significant for step 6 (aOR = 2.11; 95% CI 1.48–3.01) and step 10 (aOR = 1.36; 95% CI 0.92–2.00).

**Table 2:** Odds of achieving planned duration of breastfeeding by exposure to Baby-friendly Hospital Practices

	Achieved planned breastfeeding duration		
Characteristic	OR (95% CI)^a^	Model 1 aOR (95% CI)^b^	Model 2 aOR (95% CI)^c^
Step 4: Breastfeeding initiation within 1 hour			
No	1	1	1
Yes	1.31 (1.02–1.68)	1.06 (0.80–1.39)	1.09 (0.80–1.49)
Step 6: Only breastmilk given			
No	1	1	1
Yes	2.13 (1.65–2.75)	2.06 (1.49–2.84)	2.11 (1.48–3.01)
Step 7: Rooming-in			
No	1	1	1
Yes	0.98 (0.75–1.28)	0.74 (0.48–1.14)	0.93 (0.53–1.65)
Step 8: Breastfeeding on demand			
No	1	1	1
Yes	1.05 (0.77–1.41)	1.13 (0.70–1.82)	4.15 (1.09–15.89)
Step 9: No teat given			
No	1	1	1
Yes	1.90 (1.40–2.59)	1.18 (0.81–1.72)	1.36 (0.79–2.32)
Step 10: Provide information on breastfeeding support			
No	1	1	1
Yes	1.60 (1.13–2.27)	1.41 (0.98–2.02)	1.36 (0.92–2.00)

*OR* odds ratio, *aOR* adjusted odds ratio, *CI* confidence interval

^a^Unadjusted odds ratios

^b^Adjusted for other Baby-Friendly Hospital Practices

^c^Further adjusted for maternal age, maternal education, family income, length of residence in Hong Kong, returning to work, intention to exclusively breastfeed, participant breastfed, previous breastfeeding experience, partner’s infant feeding preference, parity, delivery type and study site

The proportion of participants who experienced one to six baby-friendly hospital practices is presented in Table 3. The frequency of exposure to one baby-friendly hospital practices was 3.1% while 19.8% of participants experienced all six practices. Among participants who experienced all six baby-friendly practices, a higher proportion achieved their individual breastfeeding goals. In the adjusted logistic regression analyses, there was a graded positive association between the number of baby-friendly hospital practices experienced and the odds of participants achieving their intended duration of breastfeeding. Participants who experienced 6 hospital practices were eight times more likely (aOR = 8.45; 95% CI 3.03–23.6) to achieve their planned duration of breastfeeding when compared with women who experienced one hospital practice. Finally, when compared with participants exposed to only one baby-friendly hospital practice, for each extra baby-friendly practice experienced, participants’ odds of achieving their planned duration of breastfeeding increased by approximately 20% (aOR = 1.21; 95% CI, 1.11–1.32) (data not shown). The Hosmer–Lemeshow goodness-of-fit tests of the logistic regression models ranged from 0.39 to 0.40, indicating a good fit for the data.

**Table 3:** Odds of achieving planned duration of breastfeeding by number of Baby-friendly Hospital Practices experienced, (*n* = 1011)

No. of Practices Experienced	All Participants No. (%)	Participants who met Intentions	Unadjusted OR (CI)	Adjusted OR (CI)^a^
1	31 (3.1)	8 (1.7)	1	1
2	185 (18.3)	75 (16.3)	1.96 (0.83–4.62)	1.93 (0.79–4.73)
3	204 (20.2)	80 (17.4)	1.85 (0.79–4.35)	2.24 (0.89–5.61)
4	178 (17.6)	81 (17.7)	2.40 (1.02–5.66)	3.35 (1.29–8.71)
5	213 (21.1)	96 (20.9)	2.36 (1.01–5.51)	4.28 (1.59–11.55)
6	200 (19.8)	119 (25.9)	4.22 (1.80–9.91)	8.45 (3.03–23.60)

*OR* odds ratio, *aOR* adjusted odds ratio, *CI* confidence interval

^a^Adjusted for maternal age, maternal education, family income, length of residence in Hong Kong, returning to work, intention to exclusively breastfeed, participant breastfed, previous breastfeeding experience, partner’s infant feeding preference, parity, delivery type and study site

## Discussion

This study is the first to examine the effect of exposure to the baby-friendly hospital practices on mothers’ achievement of their planned duration of breastfeeding in a non-Western population. In this study, we identified key maternal characteristics that were strongly related to just over half of the participants who were able to achieve participant’s individual goals. Educated mothers with previous breastfeeding experience and those living in Hong Kong for a shorter period of time were more likely to achieve participant individual goals. These characteristics were also frequently associated with a longer breastfeeding duration in other studies [17, 18]. Although these are non-modifiable characteristics, additional breastfeeding support should be provided to less educated first-time mothers both during the postnatal hospitalization and after hospital discharge to help mothers achieve their individual breastfeeding goals. Also, because breastfeeding duration with a first child is a strong predictor of breastfeeding duration with subsequent children [19], providing adequate breastfeeding support to first time mothers can be beneficial beyond that pregnancy. In contrast to other studies [20, 21] we found that returning to work was not associated with participants achieving their individual goals. However other studies have shown that return to work is associated with shorter duration of breastfeeding [22] and that maternity leave duration and full or part time work status are associated with US mothers’ ability to meet breastfeeding intentions [23]. A local study shows that approximately 85% of employed new mothers return to full-time employment before ten weeks postnatal and 88% work 40 h per week or more [24]. Therefore, most pregnant women make plans about returning to work before giving birth and their ability to combine breastfeeding and employment is likely reflected in their breastfeeding goals.

Our findings also show that baby-friendly hospital practices were strongly associated with mothers’ achievement of their intended breastfeeding duration. Step 6, giving newborns only breast milk almost doubled a mother’s likelihood of achieving her planned duration of breastfeeding. This finding is consistent with previous studies conducted in the US on the effect of in-hospital exclusive breastfeeding on mothers’ achievement of their individual breastfeeding goals [11, 25, 26]. In this sample however, even among participants who achieved their planned duration of breastfeeding, only 36% exclusively breastfed while in the hospital. Unfortunately, in-hospital formula supplementation of healthy breastfeeding infants is all too common [27, 28] and rarely medically indicated [29]. Numerous studies have shown that early formula supplementation directly undermines the initiation and duration of breastfeeding [18, 27, 30]. In 2006, Hong Kong adopted a policy that all public and private hospitals have to purchase infant formula at market price as a step toward becoming more baby-friendly. This study reinforces previous studies that early supplementation affects the initiation and duration of breastfeeding. Our study extends this finding that it helps mothers to achieve their planned duration of breastfeeding in the long term. Early breastfeeding initiation after birth, minimizing separation between mother and baby, and support and advice to minimize maternal anxiety over perceived insufficient milk supply can help to reduce unnecessary formula supplementation during the postnatal hospital stay [31]. Our findings differ from previously published findings in the US population that step 10, providing mothers with information on breastfeeding support before hospital discharge was associated with 36% increased odds of participants achieving their planned duration of breastfeeding. Many new mothers report that after going home from the hospital they feel alone and isolated and have to figure out how to breastfeed on their own [32]. Therefore among Chinese population, providing clear information to new mothers about where to find breastfeeding support may help mothers achieve their planned duration of breastfeeding and assist them to find support if needed.

Overall, each of the baby-friendly steps is highly interconnected with the others, structurally and physiologically, and the steps are statistically correlated with each other. Although only two of the six measured baby-friendly practices were individually associated with participants achieving their planned duration of breastfeeding, there was a clear dose-response pattern and exposure to increasing numbers of baby-friendly practices was associated with increasingly higher odds of participants reaching their individual goals. Participants who experienced six baby-friendly hospital practices were four and one-half times more likely to achieve their planned duration of breastfeeding when compared with women who experienced only one practice. Unfortunately, only 19.8% of our participants experienced all six baby-friendly practices. Encouragingly, since our study was conducted, two of our four study hospitals have been officially designated as baby-friendly and the other two are progressing toward baby-friendly status. Full implementation of the BFHI improves hospital practices, reduces unnecessary infant formula supplementation, and improves breastfeeding rates [33, 34].

.Strengths of this study were the minimal study attrition and the follow-up data on 97.6% of the study sample. Weaknesses of this study were that despite the large sample size, participant recruitment was not population based. Therefore, new mothers with more breastfeeding confidence may have been more likely to participate in our study, and we do not have data on eligible mothers who declined to participate. Furthermore, participants self-reported breastfeeding data in this study and therefore it may be subject to recall bias. However, other studies have shown that maternal reports of breastfeeding duration are accurate for many years after women have stopped breastfeeding [35].

## Conclusions

Findings from this study show that while a majority of new mothers fail to achieve their individual breastfeeding goals, exposure to a greater number of baby-friendly hospital practices substantially increases their odds of achieving their planned duration of breastfeeding. Broader implementation of BFHI and the provision of more breastfeeding support in the early postnatal period is also necessary to help mothers’ breastfeed for longer.

## Data Availability

The data generated and analysed during the current study are not publicly available yet.

## Abbreviations

BFHI: Baby-Friendly Hospital Initiative
aOR: Adjusted Odds Ratio
CI: Confidence Interval
